# Disparities in Outcomes following Admission for Cholangitis

**DOI:** 10.1371/journal.pone.0059487

**Published:** 2013-03-29

**Authors:** Julia McNabb-Baltar, Quoc-Dien Trinh, Alan N. Barkun

**Affiliations:** 1 Division of Gastroenterology, McGill University, Montreal, Canada; 2 Department of Surgery, Henry Ford Health System, Detroit, Michigan, United States of America; 3 Cancer Prognostics and Health Outcomes Unit, University of Montreal Health Center, Montreal, Canada; University of Washington, United States of America

## Abstract

**Introduction:**

Few have examined determinants of adverse outcomes in patients presenting with ascending cholangitis. The objective of this study was to examine factors associated with in-hospital mortality, prolonged length of stay (LOS) and increased hospital charges (HC) in patients presenting with acute cholangitis.

**Methods:**

Within the Health Care Utilization Project Nationwide Inpatient Sample (NIS), we focused on patients, 18 years and older, admitted to the emergency department with cholangitis as primary diagnosis (1998–2009). Models were fitted to predict likelihood of in-hospital mortality, prolonged LOS and increased HC. Covariates included race, day of admission, insurance status, socio-economical status and other patient and hospital characteristics.

**Results:**

Overall, weighted estimates of 248,942 patients were admitted with acute cholangitis between 1998 and 2009, of which 13,534 (5.4%) died during the admission. Multivariable analyses revealed that relative to Caucasian patients, African American, Hispanic and Asian and Pacific Islander patients were more likely to die (OR = 1.61, p<0.001, OR = 1.20, p = 0.01 and OR = 1.26, p = 0.008), to experience a prolonged LOS (OR = 1.77, p<0.001, OR = 1.30, p<0.001, 1.34, p<0.001), and to incur high HC (OR = 1.83, p<0.001, OR = 1.51, p<0.001, OR = 1.56, p<0.001). Moreover, Medicaid and Medicare patients were more likely to die (OR = 1.64, p<0.001, OR = 1.24, p<0.001), to experience a prolonged LOS (1.74, p<0.001, OR = 1.25, p<0.001) and to incur high HC (OR = 1.23, p = 0.002, OR = 1.12, p = 0.002) compared to privately insured patients. In subgroup analysis, there were no differences for Medicare patients age 65 years and over. However, those under 65, most of whom have disability or end stage renal disease, were more likely to experience the negative outcomes.

**Conclusion:**

Race and insurance status represent independent predictors of in-hospital mortality and adverse outcomes in patients presenting with cholangitis. Whether these disparities are due to biological predisposition or unequal quality of care requires further investigation. Regardless, efforts should be made to reduce these outcome disparities.

## Introduction

Ascending cholangitis is a systemic infection caused by obstruction of the biliary tract. [1] Initially described by Charcot in 1877, the treatment of this condition consists of early empirical antibiotherapy, as well as endoscopic, percutaneous or surgical drainage of the biliary tree. Mortality from this condition is low when prompt antibiotherapy and drainage are initiated [2]. However, in a case series of 145 patients, 30-day mortality when bacteremia was present remained high, approximating 11%; in that series, independent predictors of mortality included high Charlson comorbidity index (CCI), septic shock, acute renal failure, high direct bilirubinemia and malignant obstruction. [3] As highlighted in the 2007 Tokyo Guidelines, meticulous care should be given to the few patients presenting with Reynolds’ pentad [4] (confusion, hypotension, jaundice, fever and right upper quadrant abdominal pain), as the mortality rate in these cases is as high as 50% [2].

Several factors have been associated with adverse outcomes during hospitalization. Indeed, racial backgrounds[5]–[7], admission during the weekend[8]–[11], non-private insurance status[12]–[14] have been linked with increased mortality, complications, prolonged length of stay and increased costs. To our knowledge, the effect of these variables has never been examined in the context of ascending cholangitis.

Based on these considerations, we explore patient characteristics and hospital factors associated with in-hospital mortality and, secondarily, with prolonged length of stay and high hospital charges. Our analysis relies on a large contemporary (1998–2009) population-based cohort of individuals admitted from the emergency department with cholangitis.

## Materials and Methods

### Ethics

Institutional review board approval was not needed because this study did not involve the analysis of human subjects; a waiver was obtained from the University of Montreal Institutional Review Board. Since this study involved the analysis of a population-based dataset, written consent by patients was not required.

### Data Source

Data from 1998 to 2009 of the Nationwide Inpatient Sample (NIS) were abstracted. The NIS includes inpatient discharge data collected via federal-state partnerships, as part of the Agency for Health care Research and Quality’s Health care Cost and Utilization Project (HCUP). As of the year 2009, the NIS contained administrative data on approximately 8 million hospital stay each year from 1050 hospitals within 40 states, approximating 20% of community hospitals within the United States, including public hospitals and academic medical centers. The NIS is the sole hospital database in the United States with charge information on all patients regardless of payer, including persons covered by Medicare, Medicaid, private insurance, and the uninsured. This study was exempt from institutional review board approval in accordance with provincial and federal legislation when dealing with population-based publicly available data.

### Sample Population

Using the International Classification of Diseases 9^th^ Revision, Clinical Modification (ICD-9-CM), all patients (18 years of age and older) with a primary diagnosis of cholangitis (ICD-9-CM code 576.1) admitted non-electively in the emergency department were considered for the study. Hospital sampling weights were used to estimate the total number of patients presenting with cholangitis in the USA, yielding a weighted national estimate of 248,942 cases.

### Baseline Patient and Hospital Characteristics

For all patients, the following variables were available: age, gender, race (Caucasian, African American (AA), Hispanic, Asian and Pacific Islander [15], Native American, other or missing), CCI, day of admission (weekend, weekday), insurance status (Private, Medicare, Medicaid, Uninsured and other), socio-economic status, annual hospital caseload (AHC), as well as hospital region, location and teaching status. Information about hospital region was obtained from the American Hospital Association Annual Survey of Hospitals, and defined by the United States Census Bureau [16]. The CCI was derived from ICD-9 codes according to previously established criteria [17] and was stratified according to four levels: 0, 1, 2 and ≥3. Socioeconomic status was derived from median zip code income and was stratified according to quartiles; very-low, low, high, very-high. AHC was defined according to the number of patients admitted with cholangitis at each participating institution during each study calendar year. Hospitals were divided into caseload quartiles named very-low, low, high, very-high, defined as < = 8, 9–15, 16–27 and >28. Hospitals were dichotomized into academic and non-academic institutions. The hospital’s academic status was obtained from the AHA Annual Survey of Hospitals. A hospital is considered to be a teaching hospital if it has an American Medical Association (AMA)-approved residency program, is a member of the Council of Teaching Hospitals or has a ratio of full-time equivalent interns and residents to beds of 0.25 or higher.

### Procedures Performed during Hospitalization

Procedures including diagnostic and therapeutic Endoscopic Retrograde Cholangiopancreatography (ERCP) (ICD-9-CM codes 51.10, 51.11, 52.13, 51.14, 51.64, 51.81, 51.85, 51.86, 51.87, 51.88, 51.99, 97.05) percutaneous transhepatic drainage with or without stone extraction (ICD-9-CM code 51.98, 51.96), surgical drainage (ICD-9-CM code 51.41, 51.42, 51.43, 51.49, 51.51, 51.59) and cholecystectomy (ICD-9-CM code 51.2, 51.21, 51.22, 51.23, 51.24) were assessed.

### Etiology of the Biliary Obstruction

The etiology of the biliary obstruction was stratified as choledocholithiasis (ICD-9-CM code 574.x), neoplastic process, i.e. cholangiocarcinoma, pancreatic cancer, gallbladder carcinoma, ampullary carcinoma and other (ICD-9-CM codes 155.1, 156.0, 156.1, 156.2, 156.8, 156.9, 157.0, 157.1, 157.2, 157.3, 157.4, 157.8, 157.9, 230.8, 235.3) and unknown or other etiology of obstruction (ICD-9-CM code 576.2, 576.9, 751.61).

### In-hospital Mortality, Length of Stay and Hospital Charges

In-hospital mortality information is coded from disposition of patient. Length of stay, provided by the NIS, is calculated by subtracting the admission date from the discharge date. Prolonged length of stay was defined as a hospital stay beyond the 75^th^ percentile of 10 days (LOS). High hospital charges was defined as charges above the 75^th^ percentile of 46,740$ (HC). Patients with missing or invalid length of stay, hospital charges and in-hospital mortality status were not considered within the current study nor were patients transferred to another facility.

### Statistical Analysis

Descriptive statistics focused on frequencies and proportions for categorical variables. Means, medians and ranges were reported for continuously coded variables. For continuous variables, normality was assessed using the Kolmogorov-Smirnov test [18], the Shapiro-Wilk test [15] and graphical plots. As continuous variables were not normally distributed, univariate comparisons were made using the Mann-Whitney test. Chi-square tests were used to compare the statistical significance of differences in proportions.

We used multivariable logistic regression models to adjust for confounding. Three models were created using in-hospital mortality, LOS, and HC in excess of the 75^th^ percentile as dependent variables while adjusting for the effect of race, day of admission, insurance status, socio-economic status and other patient and hospital characteristics. Within each model, generalized estimating equations (GEEs) adjusted for clustering within hospitals. Moreover, in the cohort, 20.9% of patients had missing race data. As per HCUP recommendations, these patients are included in our analyses to allow the reporting of nationally representative figures. However, since it represents a significant number of patients, we performed sensitivity analyses by excluding patients of missing race in the multivariable models to ensure the absence of bias. Our working hypothesis was that the method of drainage did not influence the outcomes of cholangitis. Nonetheless, to ensure that the type of treatment was not a confounder, we performed sensitivity analysis by including treatment type as an independent variable in the multivariable models. Furthermore, given that most patients 65 years of age or older have Medicare coverage, we conducted stratified sub-analyses for patients <65 and ≥65 years. All tests were two-sided with a statistical significance set at *P* = 0.01. Analyses were conducted using the R statistical package (the R foundation for Statistical Computing, version 2.15.1).

## Results

### Study Sample, Procedures, and Outcomes

Between 1998 and 2009, a weighted estimate of 248,942 patients was admitted with cholangitis. Overall, 5.4% of patients died during admission. The weighted rates of ERCP, percutaneous biliary drainage, surgical biliary drainage, cholecystectomy, prolonged LOS, and HC stratified according to outcome are presented in Table 1. Patients who died during hospitalization were more likely to experience a prolonged LOS (44.1 vs. 22.8%, *P*<0.001) and to incur higher HC (53.3 vs. 26.3, *P*<0.001) Furthermore, patients who died during hospitalized were less commonly treated with ERCP (32.2 vs. 53.8%, *P*<0.001) but more commonly treated with surgical and percutaneous drainage (6.6, 6.9 vs. 4.9 and 4.1%, *P*<0.001). Finally, cholecystectomies were less commonly performed in patients with in-hospital mortality (12.5 vs. 21.5%, *P*<0.001).

**Table 1:** Weighted outcomes and interventions during hospitalization according to mortality.

	Alive upon discharge (%)	In-Hospital mortality (%)	*P*
**No. of patients**	235,408	13,534	–
**Prolonged length of stay**	22.8	44.1	<0.001
**High hospital charges**	26.3	53.3	<0.001
**ERCP**	53.8	32.3	<0.001
**Percutaneous drainage**	4.1	6.9	<0.001
**Surgical drainage**	4.9	6.6	<0.001
**Cholecystectomy**	21.5	12.5	<0.001

**Abbreviation:** ERCP : Endoscopic Retrograde Cholangiopancreatography.

### Demographics and Clinical Characteristics

Patients who died during hospitalization were older (median age 73 vs. 70 years, *P*<0.001) and had more comorbidities (CCI≥3 97.2 vs. 90.3%, *P*<0.001). Higher rates of in-hospital mortality were recorded in AA, Hispanic and API patients, compared to Caucasian patients (7.7, 5.5, 7.1 vs. 5.4%, *P*<0.001). ERCP was less performed in AA than Caucasians (45.9 vs. 53.1%, *P*<0.001), but more in API and Hispanics than Caucasians (59.3% and 59,5% vs. 53.1%, *P*<0.001). Moreover, higher rates of in-hospital mortality were recorded in Medicare and Medicaid patients relative to privately insured patients (6.6, 5.4 vs. 3.2%, *P*<0.001). Weekend admission was associated with decreased in-hospital mortality compared to weekday admission (4.8 vs. 5,7%, *P*<0.001). Bile duct obstruction by neoplasm was associated with higher mortality compared to obstruction by choledocholithiasis (8.3% vs. 3.8%, *P*<0.001). A higher proportion of patients who died during hospitalization were treated at high AHC (26.5 vs. 23.4%, *P*<0.001), urban (90.3 vs. 87.7%, *P*<0.001) and teaching (51.0 vs. 46.4%, *P*<0.001) hospitals. Other demographic characteristics are listed in Table 2.

**Table 2:** Weighted demographic characteristics of patients admitted with cholangitis, stratified according to in-hospital mortality, Nationwide Inpatient Sample, 1998–2009.

	Alive upon discharge (%)	In-hospital mortality (%)	*P*
No. of patients	235,408 (94.6)	13,534 (5.4)	
**Median age**	70	73	<0.001
**(25^th^, 75^th^ percentile [IQR])**	(54, 80 [26])	(64, 84 [20])	
**Gender:** Male	49.5	50.8	0.003
Female	50.5	49.2	
**CCI** † **:** 0	6.3	1.6	<0.001
1	2.8	0.8	
2	0.7	0.3	
≥3	90.3	97.2	
**Race:** Caucasian	54.8	53.9	<0.001
African American	6.4	9.3	
Hispanic	10.2	10.4	
Asians and Pacific Islanders	5	6.6	
Native American	0.3	0.3	
Other	2	2	
Unknown	21.3	17.6	
**Socio-Economic:** Very-low	15.9	17.7	<0.001
Low	22.8	22.2	
High	25.5	24.6	
Very-high	33.4	33.2	
Unknown	2.4	2.3	
**Day of admission:** Weekday	72.1	75.1	<0.001
Weekend	27.7	24.5	
Unknown	0.2	0.3	
**Insurance:** Private	27.8	15.9	<0.001
Medicaid	8.6	8.5	
Medicare	57.2	70.7	
Other	6.4	4.9	
**Diagnostic:**Choledocholithiasis	47	31.9	<0.001
Neoplasm	10.2	16.1	
Unknown	42.9	52	
**AHC:** Very-low	28.1	26.2	<0.001
Low	23.7	24.6	
High	24.8	22.7	
Very-high	23.4	26.5	
**Hospital location:** Rural	12.3	9.7	<0.001
Urban	87.7	90.3	
**Hospital region** ‡ **:** Northeast	23.7	28.5	<0.001
Midwest	18	14.3	
South	30.8	28.3	
West	27.2	28.9	
**Institutional academic** **status**			<0.001
Non-teaching	53.6	49	
Teaching	46.4	51	

**Abbreviation:** CCI: Charlson Comorbidity Status, IQR: interquartile range, AHC: Annual Hospital Caseload.

† Based on Comorbidity developed by Charlson et al. and adapted by Deyo et al.

‡ Hospital region is defined by the US Census Bureau.

### Multivariable Analyses

Multivariable logistic regression adjusted for clustering (Table 3) revealed that relative to Caucasian patients, AA, Hispanic and API patients admitted with cholangitis were more likely to die (OR = 1.61, *P*<0.001, OR = 1.20, *P* = 0.010 and OR = 1.26, *P* = 0.008), to experience a more prolonged LOS (OR = 1.77, *P*<0.001, OR = 1.30, *P*<0.001, OR = 1.34, *P*<0.001) and to incur increased HC (OR = 1.83, *P*<0.001, OR = 1.51, *P*<0.001, OR = 1.56, *P*<0.001). Separate multivariable analyses excluding patients with unknown race were performed and the findings were similar (data not shown). Furthermore, Medicaid and Medicare insured patients were at increased risk of in-hospital mortality (OR = 1.64, *P*<0.001, OR = 1.24, *P* = 0.001), prolonged LOS (OR = 1.74, *P*<0.001, OR = 1.25, *P*<0.001) and increased HC (OR = 1.23, *P* = 0.002, OR = 1.12, *P* = 0.002), relative to privately insured patients. Moreover, increasing patient age and high CCI were independent predictors of in-hospital mortality (OR = 1.03, *P*<0.001, OR = 5.41, *P*<0.001), prolonged LOS (OR = 1.01, *P*<0.001, OR = 3.60, *P*<0.001), and high HC (OR = 1.01, *P*<0.001, OR = 2.63, *P*<0.001). Bile duct obstruction by neoplasm was associated with higher mortality (OR = 2.47, *P*<0.001) and prolonged LOS (OR = 1.30, *P*<0.001) compared to obstruction due to lithiasis. Weekend admission was associated with lowered in-hospital mortality (OR = 0.87, *P* = 0.003) and high HC (OR = 0.92, *P*<0.001), relative to weekday admission. Finally, higher socioeconomic status was associated with reduced odds of a prolonged length of stay (OR = 0.88, *P* = 0.001). Separate multivariable analyses that included treatment type as independent variable demonstrated the robustness of our main findings (data not shown).

**Table 3:** Multivariable logistic regression analyses with general estimation equation adjustment assessing in-hospital mortality, prolonged length of stay and high hospital charges, Nationwide Inpatient Sample, 1998–2009.

	In-hospital mortality		Prolonged length of stay		High hospital charges	
Variables	Odds ratio (95% confidence interval)	*P*	Odds ratio (95% confidence interval)	*P*	Odds ratio (95% confidence interval)	*P*
**Age**	1.03 (1.03–1.04)	<0.001	1.01 (1.01–1.02)	<0.001	1.01 (1.01–1.01)	<0.001
Gender						
Male	Ref.		Ref.		Ref.	
Female	0.92 (0.84–1.00)	0.041	0.91 (0.87–0.95)	<0.001	0.86 (0.82–0.90)	<0.001
**Race**						
White	Ref.		Ref.		Ref.	
Black	1.61 (1.38–1.87)	<0.001	1.77 (1.63–1.93)	<0.001	1.83 (1.62–2.07)	<0.001
Hispanic	1.20 (1.05–1.37)	0.01	1.30 (1.19–1.43)	<0.001	1.51 (1.32–1.72)	<0.001
Asian	1.26 (1.06–1.50)	0.008	1.34 (1.19–1.53)	<0.001	1.56 (1.27–1.91)	<0.001
Native American	1.31 (0.64–2.66)	0.461	1.44 (1.02–2.04)	0.039	0.99 (0.66–1.47)	0.94
Other	1.11 (0.83–1.51)	0.476	1.60 (1.39–1.85)	<0.001	1.68 (1.42–1.99)	<0.001
Unknown	0.99 (0.87–1.13)	0.864	1.00 (0.92–1.08)	0.99	0.64 (0.55–0.74)	<0.001
**Charlson score** †						
0	Ref.		Ref.		Ref.	
1	0.98 (0.58–1.67)	0.944	1.01 (0.80–1.27)	0.933	0.97 (0.78–1.21)	0.796
2	1.60 (0.79–3.22)	0.193	1.48 (1.05–2.09)	0.027	1.26 (0.88–1.79)	0.213
≥3	5.41 (3.94–7.41)	<0.001	3.60 (3.14–4.13)	<0.001	2.63 (2.27–3.06)	<0.001
**Diagnosis**						
Stone	Ref.		Ref.		Ref.	
Neoplasm	2.47 (2.17–2.80)	<0.001	1.30 (1.21–1.40)	<0.001	0.96 (0.89–1.05)	0.361
Unknown	2.57 (2.36–2.80)	<0.001	0.99 (0.94–1.05)	0.783	0.74 (0.70–0.79)	<0.001
**Socio-Economic**						
Very-low	Ref.		Ref.		Ref.	
Low	0.91 (0.81–1.03)	0.147	0.96 (0.90–1.04)	0.303	0.97 (0.88–1.07)	0.552
High	0.87 (0.77–0.99)	0.033	0.89 (0.83–0.96)	0.003	0.90 (0.81–1.01)	0.063
Very-high	0.86 (0.76–0.97)	0.016	0.88 (0.82–0.95)	0.001	1.07 (0.93–1.22)	0.361
Unknown	0.90 (0.69–1.17)	0.413	0.99 (0.84–1.15)	0.845	0.94 (0.78–1.13)	0.531
**Insurance**						
Private	Ref		Ref		Ref	
Medicaid	1.64 (1.37–1.96)	<0.001	1.74 (1.58–1.92)	<0.001	1.23 (1.08–1.40)	0.002
Medicare	1.24 (1.09–1.40)	0.001	1.25 (1.17–1.33)	<0.001	1.12 (1.04–1.20)	0.002
Other	1.47 (1.19–1.82)	<0.001	1.35 (1.20–1.51)	<0.001	1.00 (0.88–1.15)	0.958
**Annual Hospital Caseload**						
Very-low	Ref.		Ref.		Ref.	
Low	1.02 (0.90–1.14)	0.808	1.07 (1.00–1.15)	0.058	1.08 (0.97–1.21)	0.148
High	0.85 (0.76–0.96)	0.011	0.99 (0.92–1.07)	0.861	1.18 (1.02–1.36)	0.026
Very-high	0.96 (0.83–1.12)	0.624	1.07 (0.96–1.19)	0.231	1.30 (1.05–1.60)	0.015
**Hospital location**						
Rural	Ref.		Ref.		Ref.	
Urban	1.28 (1.09–1.50)	0.003	1.80 (1.61–2.00)	<0.001	3.30 (2.73–4.01)	<0.001
**Academic status**						
Non-teaching	Ref.		Ref.		Ref.	
Teaching	1.19 (1.08–1.31	0.001	1.13 (1.05–1.21)	0.001	1.09 (0.91–1.30)	0.348
**Hospital Region** ‡						
Northeast	Ref.		Ref.		Ref.	
Midwest	0.74 (0.62–0.88)	0.001	0.70 (0.63–0.78)	<0.001	0.88 (0.73–1.07)	0.190
South	0.81 (0.72–0.92)	0.001	0.85 (0.78–0.93)	0.001	1.16 (0.98–1.39)	0.09
West	0.95 (0.84–1.08)	0.435	0.67 (0.60–0.74)	<0.001	2.62 (2.07–3.32)	<0.001

† Based on Comorbidity developed by Charlson et al. and adapted by Deyo et al.

‡ Hospital region is defined by the US Census Bureau.

The results of sub-analyses stratified according to age groups (<65 and > = 65) are displayed in Table 4. In patients younger than 65 years, Medicaid and Medicare coverages were independent predictors of poor outcomes for all three endpoints examined. Conversely, in the subset of patients 65 years or above, Medicare was not an independent predictor of any of the three outcomes, although a trend towards significance existed for prediction of high hospital charges (OR = 1.13, p = 0.053).

**Table 4:** Multivariable logistic regression analyses with general estimation equation adjustment assessing in-hospital mortality, prolonged length of stay and high hospital charges stratified according to age (<65 years and ≥65 years), Nationwide Inpatient Sample, 1998–2009.

Patients younger than 65 years									
	In-hospital mortality			Prolonged length of stay			High hospital charges		
Variables	Odds ratio (95% confidence interval)		*P*	Odds ratio (95% confidence interval)		*P*	Odds ratio (95% confidence interval)		*P*
**Race**									
White	Ref.			Ref.			Ref.		
Black	1.57 (1.24–1.99)		<0.001	1.72 (1.53–1.95)		<0.001	1.85 (1.59–2.15)		<0.001
Hispanic	1.28 (0.98–1.67)		0.068	1.27 (1.11–1.46)		0.001	1.51 (1.29–1.76)		<0.001
Asian	1.01 (0.69–1.49)		0.950	1.02 (0.84–1.25)		0.818	1.38 (1.09–1.74)		0.007
Native American	1.16 (0.30–4.39)		0.832	1.37 (0.83–2.26)		0.217	0.82 (0.44–1.53)		0.529
Other	1.39 (0.84–2.33)		0.203	1.52 (1.20–1.93)		<0.001	1.71 (1.35–2.16)		<0.001
Unknown	0.96 (0.76–1.22)		0.743	1.00 (0.88–1.12)		0.935	0.66 (0.55–0.79)		<0.001
**Insurance**									
Private	Ref.			Ref.			Ref.		
Medicaid	1.89 (1.51–2.37)		<0.001	1.86 (1.65–1.09)		<0.001	1.25 (1.07–1.45)		0.004
Medicare	1.88 (1.53–2.30)		<0.001	1.88 (1.68–2.104)		<0.001	1.36 (1.21–1.53)		<0.001
Other	1.55 (1.18–2.04)		0.002	1.46 (1.28–1.66)		<0.001	1.05 (0.90–1.22)		0.549
**Patients 65 years or older**									
**Race**									
White	Ref.			Ref.			Ref.		
Black	1.58 (1.29–1.92)	<0.001		1.87 (1.64–2.12)	<0.001		1.93 (1.64–2.27)	<0.001	
Hispanic	1.21 (1.01–1.44)	0.036		1.31 (1.17–1.47)	<0.001		1.50 (1.29–1.76)	<0.001	
Asian	1.39 (1.15–1.68)	0.001		1.43 (1.21–1.68)	<0.001		1.52 (1.18–1.95)	0.001	
Native American	1.60 (0.69–3.71)	0.274		1.83 (1.16–2.89)	0.009		1.33 (0.79–2.24)	0.292	
Other	1.07 (0.74–1.56)	0.715		1.60 (1.34–1.92)	<0.001		1.55 (1.24–1.94)	<0.001	
Unknown	0.99 (0.85–1.15)	0.908		0.99 (0.90–1.09)	0.794		0.60 (0.51–0.71)	<0.001	
**Insurance**									
Private	Ref.			Ref.			Ref.		
Medicaid	1.34 (0.96–1.86)	0.082		1.69 (1.41–2.04)	<0.001		1.38 (1.11–1.73)	0.005	
Medicare	1.16 (0.96–1.40)	0.138		1.07 (0.96–1.19)	0.219		1.13 (1.00–1.28)	0.053	
Other	1.64 (1.13–2.38)	0.010		1.09 (0.85–1.39	0.499		1.04 (0.80–1.35)	0.773	

## Discussion

Previous large-scale population-based reports have identified several patient and system attributes, such as race, insurance status and day of admission, associated with adverse outcomes of hospitalization for various medical conditions. For example, AA patients are at increased risk of in-hospital mortality following hepatectomy and cholecystectomy [5], [6]. Medicaid patients are at increased risk of postoperative complications and mortality following colorectal surgery [14]. Finally, weekend admission for acute upper-gastrointestinal bleed [8] and stroke [10] are associated with increased mortality relative to weekday admission. To our knowledge, the effect of these parameters on outcomes of patients admitted with cholangitis has never been assessed. Based on these considerations, we examined the effect of several important variables on in-hospital mortality, prolonged LOS and increased HC in a large contemporary (1998–2009) population-based cohort of individuals admitted with ascending cholangitis.

Our results demonstrated several important points. First, we identified key differences in treatment delivery between patients who died and those who did not. Indeed, lower rates of ERCP and cholecystectomy were recorded in patients who died during hospitalization. Conversely, these patients experienced higher rates of percutaneous and surgical drainage. The Tokyo 2007 guidelines recommend ERCP as the standard of care for drainage when possible [2]. These findings may suggest that this subset of patients may have been were sicker at presentation, perhaps preventing them from undergoing a timely ERCP [19]. Moreover, the overall rate of biliary drainage is lower that expected, likely because the NIS only contains data on procedures performed during admission and does not account for elective interventions.

Second, our results indicate that on average, worse outcomes, including in-hospital mortality, prolonged LOS and increased HC apply to AA, Hispanic and API patients admitted with cholangitis. Several hypotheses may be proposed to explain our findings. Indeed, it is possible that AA, Hispanic and API patients present late with more severe disease and therefore worsening outcomes. Previous reports have documented racial differences in stage and severity of disease at presentation [20]. Second, anatomical or biological differences may explain the disparities. For example, Asians are at increased risk of bile duct injury during cholecystectomy [21], purportedly due to a higher degree of anatomical variations. It is also possible that, when all confounders are accounted for, AA, Hispanics and API do not receive the same level of care as their Caucasian counterparts as has been suggested previously [12], [13], [22]. It is possible that barriers, including language barriers [23], contribute to racial disparities. Furthermore, it is possible that disparities in access to ERCP may be responsible for the variation in outcomes. While we did find differences in ERCP rates across the racial groups, in two of three cases they were significantly higher than for Caucasians and therefore not likely to have been a major factor in explaining the mortality differences. Finally, it has been suggested by some that a wide range of patient-physician relationship issues may also in part explain the observed disparities [24], [25].

Furthermore, our study also shows that Medicaid and Medicare patients are at increased risk of in-hospital mortality, prolonged LOS and increased hospital charges compared to their privately insured counterparts. Several hypotheses may be postulated to explain the discrepancy. Medicaid and Medicare patients may present with later stage of disease, as reported in other conditions [26], [27]. Poor access to health care may also be at cause [28]. In addition, the difference in outcomes may also be due to unaccounted socioeconomic differences, as median zip code income does not entirely approximate socioeconomic status. Finally, subset analyses demonstrate that the effect of Medicare coverage on adverse outcomes is more pronounced in patients younger than 65 years of age, suggesting the presence of unmeasured confounders, since Medicare eligibility under 65 is restricted to individuals with long-term disabilities or those diagnosed with specific diseases. It is also possible that the difference observed in Medicare patients 65 and above may be overstated. Indeed, in future studies, Medicare patients younger than 65 years of age might be used as a marker of comorbidities, in addition to CCI.

In this study, weekend admission was associated with a decreased risk of in-hospital mortality and increased hospital charges. These findings contrast with previous studies reporting worse outcomes in patients admitted during the weekend [8]–[11], presumably due to a lack of resources and staffing on weekends. Yet, it is possible that weekend patients may receive more prompt attention from the medical team, who might not have been readily available during regular work hours, as has been suggested for other conditions. [29] [30] Indeed, Carr *et al*. observed similar findings in the trauma population where weekend admission was associated with decreased mortality [29]. Similarly, Luyt *et al.* observed a reduction in the mortality of patients in the intensive care unit during off hours [30].

Finally, several patient and hospital characteristics were also associated with worse outcomes. These include increasing age and CCI. Indeed, we corroborate previous reports that these two attributes were associated with higher in-hospital mortality [3], prolonged LOS and high HC [31], [32]. Biliary obstruction due to neoplasm was also associated with worse outcomes relative to choledocholithiasis, as also previously reported [1], [3]. Conversely, higher socio-economic status was associated with shorter LOS, which was also recorder in a population-based study from Belgium [33]. Patients treated at urban and teaching hospitals were at increased risk of in-hospital mortality, prolonged LOS and increased HC, as seen in other conditions [34], [35]. Higher in-hospital mortality recorded at high-volume, urban and/or teaching institutions may be due to differences in referral and transfer patterns, consequently case-mix. For example, data from the Washington State Commission Hospital Abstract Reporting System has shown that longer travel distance is associated with more resources and higher hospital charges [36]. Finally, the association between in-hospital mortality and geographic variation does not follow other reports, as northeastern location was associated with poorer outcomes in our analysis. Explanatory factors for this discrepancy are unclear and may relate to unadjusted confounding, unrecognized biological factors or a type I error.

Limitations include the study design; indeed, observational studies cannot be used as proof of a causal relationship. Some reasons for this are the inability to adjust for important patient variables such as disease characteristics, personal preferences, education, and disease severity. Unavailability of individual gastroenterologist, surgeon, and interventional radiologist volume represents another limitation, which is shared by several other analyses [14], [37]. It is also possible that the true mortality is underestimated as some patients may have died at other institutions where their mortality was not captured. Moreover, the accuracy of administrative ICD-9-CM claims for identification of cholangitis and ERCP has never been validated within the NIS, which could lead to some degree of misclassification. Finally, since our analyses are based on a relatively large dataset, it is important to noteworthy that some of our results might be statistically significant, but of low clinical yield. Nonetheless, most of the differences discussed in the current manuscript have important health policy and clinical implications.

To summarize, in patients presenting with cholangitis, race and insurance status represent independent predictors of in-hospital mortality. Specifically, AA, Hispanic, API patients presenting with cholangitis are at increased risk of in-hospital mortality, prolonged LOS and high hospital charges, relative to their Caucasian counterparts. Moreover, Medicaid and Medicare patients are also at increased risk of in-hospital mortality, prolonged LOS and high hospital charges, relative to privately insured patients. Whether these disparities are due to biological predisposition or unequal quality of care require further investigation.
